# Evaluation of Red Blood Cell Biomechanics in the Setting of Cancer‐Associated Anemia and Chemotherapy

**DOI:** 10.1111/micc.70074

**Published:** 2026-06-26

**Authors:** Deirdre Finnigan, Nick Cruickshanks, George Ilbawi, Regan Bucciol, Yousra Tera, Maha Othman

**Affiliations:** ^1^ Department of Biomedical and Molecular Sciences Queen's University Kingston Ontario Canada; ^2^ Department of Public Health Sciences Queen's University Kingston Ontario Canada; ^3^ Clinical Pathology Department, Faculty of Medicine Mansoura University Mansoura Egypt; ^4^ School of Baccalaureate Nursing St. Lawrence College Kingston Ontario Canada

**Keywords:** aggregation, anemia, deformability, elasticity, erythrocytes, hemorheology

## Abstract

**Introduction:**

Red blood cells (RBCs) possess distinct biomechanical properties that enable their survival and efficient oxygen delivery. Cancer‐associated anemia, frequently compounded by chemotherapy, is a major clinical challenge, yet little is known about how RBC biomechanics contribute to its pathophysiology. This study evaluates the biomechanical properties of RBCs in patients with cancer compared to controls and within patients before and after chemotherapy.

**Methods:**

Biomechanical properties of RBCs were assessed in 110 women with breast, ovarian, or endometrial cancer, measured before and after chemotherapy, and compared findings with 35 healthy female controls. Thirteen biomechanical parameters were assessed using the MIZAR automated rheometer.

**Results:**

Relative to controls, pre‐chemotherapy cancer patients exhibited significantly higher RBC aggregation and elasticity. Within the cancer cohort, anemic patients demonstrated more deformable and elastic RBCs, with increased aggregation compared to non‐anemic patients. Following chemotherapy, patients displayed reduced RBC deformability but further increased elasticity, consistent with chemotherapy‐induced alterations to membrane structure and function; these effects were most pronounced in anemic patients.

**Conclusion:**

We report novel rheological observations indicating that both cancer and chemotherapy are associated with alterations in RBC biomechanics, and that anemia further amplifies these changes. Importantly, cancer‐associated anemia appears to involve impaired RBC quality. Recognition of biomechanical dysfunction may provide new insights into the mechanisms of cancer‐related anemia and support the development of more comprehensive diagnostic and management strategies.

## 1 Introduction

Human erythrocytes, or red blood cells (RBCs), are critical in the delivery of oxygen to and removal of carbon dioxide from all cells and tissues of the body. To do this effectively, RBCs must possess unique characteristics to be able to withstand high levels of shear stress as they move through vessels of varying diameter without compromising their cellular membranes or integrity. These characteristics are known as rheologic properties, and include aggregability, deformability, and elasticity [1, 2]. Influenced by plasma proteins, aggregability refers to RBC clumping involving RBC‐RBC interaction, a reversible process influenced by proteins such as fibrinogen and immunoglobulins. Aggregability is inversely related to blood flow; slower flow has less disturbance on aggregates [3]. However, aggregation has been shown to enhance vascular blood flow through its effects on hemodynamic mechanisms. Deformability is the ability of RBCs to minimize flow resistance by changing their shape based on flow conditions, which is critical for effective oxygen delivery and RBC survival [4]. Any RBCs that cannot pass through splenic vessels are sequestered and destroyed [5]. Related to deformability, elasticity is the ability of RBCs to deform and recover their original shape without membrane or content damage. Elasticity is an inherent mechanical property of the cells, while deformability refers to the cell's ability to change shape, influenced by elasticity and internal factors. These unique biomechanical factors of RBCs play a critical role in microcirculation, where RBCs must traverse capillaries with diameters smaller (4–8 μm inner diameter) than RBCs themselves (7.5–8.7 μm) [6, 7]. Effective microcirculatory flow therefore depends on the ability of RBCs to deform, recover their shape, and avoid excessive aggregation in order to maintain adequate tissue perfusion and oxygen delivery.

RBCs also participate in coagulation through several mechanisms including platelet interactions, rheological effects, and platelet margination [8]. Recent evidence suggests that red cell biochemistry—such as membrane composition, ion transport, and metabolic pathways—also plays a key role in RBC function, influencing their ability to respond to mechanical stress and survive in circulation [9]. RBCs are increasingly recognized as active contributors to coagulation and thrombus structure rather than passive bystanders within clots. Through interactions with fibrin and platelets, RBCs influence clot architecture, density, and susceptibility to fibrinolysis, while altered RBC deformability and aggregation can further affect thrombus formation and stability [10]. RBC biomechanical alterations may contribute to cancer‐associated thrombosis (CAT) by altering blood viscosity, platelet margination, microcirculatory flow, and clot contraction. Changes in RBC rheologic properties from cancer and chemotherapy may impact established prothrombotic pathways involved in CAT [11].

Anemia is a common and significant complication in cancer patients, often exacerbated by both the cancer itself and its treatments [12]. Cancer‐associated anemia is associated with decreased survival and increased morbidity in patients [12, 13]. It can result from several factors, including direct effects of the malignancy on the bone marrow, nutritional deficiencies (e.g., iron and folate), and chronic inflammation associated with cancer progression [13]. Chemotherapy, while effective in treating cancer, can cause anemia via bone marrow suppression, reducing RBC production, and inducing oxidative stress that affects RBC survival [14]. Moreover, chemotherapy‐induced anemia may be further complicated by treatment‐related side effects such as gastrointestinal bleeding or erythropoietin suppression [15]. Despite the established association between anemia and cancer, the precise mechanisms linking the biomechanical properties of RBCs to cancer‐related anemia remain poorly understood. This gap in knowledge is particularly important as alterations in RBC deformability or other biomechanical properties may not only contribute to the pathophysiology of anemia but also influence treatment outcomes and patient quality of life. Furthermore, the impact of chemotherapy on RBC function, particularly in terms of biomechanical properties, is still an underexplored area.

Female health remains understudied compared to the general population, and anemia disproportionately affects women globally [16, 17]. Thus, female cancers were identified as the focus of this study, as a novel area of research. This study aims to assess the relationship between RBC biomechanical properties and anemia in female cancer patients, focusing on changes that occur during and after chemotherapy treatment. Understanding these relationships may offer new insights into the pathophysiology of cancer‐associated anemia, patients' related outcomes, and potentially inform more effective management strategies for this common and debilitating condition.

## 2 Materials and Methods

This is a prospective study that recruited 110 individuals from general oncology and gynecological oncology clinics at Kingston Health Science Centre who were diagnosed with breast, endometrial, or ovarian cancer and planned to receive chemotherapy treatments. Ethics approval was secured for the study from the Queen's University Health Sciences and Affiliated Teaching Hospitals Research Ethics Board and St. Lawrence College Research Ethics Board.

Inclusion criteria for breast cancer included all cancer patients planned to start chemotherapy (any type), good performance status (ability to carry out daily activities) as determined by their physician, with life expectancy of at least 3 months, and no current use of blood thinners. Inclusion criteria for gynecological cancers (ovarian and endometrial) included all patients starting chemotherapy before surgery (neoadjuvant) or following surgery (adjuvant), good performance status, and no current use of blood thinners. Exclusion criteria included age younger than 18 years, life expectancy less than 3 months, ongoing pregnancy (for breast cancer patients), major psychiatric disorders, recent (< 6 months) episode of venous thromboembolism (VTE) or acute coronary syndrome, active anticoagulant treatment (for any indication), and scheduled open elective curative abdominal or pelvic surgery under general anesthesia (for breast cancer patients only). Additional exclusion included those hospitalized due to stroke, acute coronary syndrome, congestive heart failure, acute respiratory failure, recent VTE on blood thinners, history of coagulation disorder, and early‐stage breast cancer treated with curative intent. Individuals with incomplete data (e.g., only one sample rather than one before and one after chemotherapy commencement) were excluded.

Demographic and clinical characteristics of the included individuals with cancer were collected, including age, BMI, self‐reported menopausal stage, smoking status, and comorbidities including diabetes, hypertension, dyslipidemia, and history of blood disorders. Cancer type, stage, metastasis, and chemotherapy data were also collected.

Blood was drawn before the start of chemotherapy and after one or two cycles of chemotherapy and was tested for complete blood count (CBC) data including hemoglobin (Hb), hematocrit (Hct), RBC and platelet count, and RBC indices. Anemia was defined according to the WHO criteria and the CTCAE (v5.0) when patients had Hb less than 12.0 g/dL and categorized into 4 grades according to severity [18]. Thirteen RBC biomechanical parameters were generated using the MIZAR automated syllectometry‐based rheometer (ALCOR Scientific, Rhode Island) and evaluated for all patients. A schematic representation of these RBC biomechanical properties is provided in Figure S1. Table S1 shows the explanation of each of the 13 parameters. Pre‐chemotherapy data were compared to post‐chemotherapy data and data from 35 healthy female controls.

The MIZAR rheometer uses a hydraulic pump to flow a blood sample within a cell. Shear stress is applied to the flow cell, while a light source and optical detector flank the cell to capture light transmission through the sample. A syllectogram is produced based on the light transmission throughout the shear stress application and removal, providing numerical data related to aggregation, deformation, and elasticity [2].

Welch's *t*‐test was used to compare means between unpaired groups and paired *t*‐test to compare means between paired groups (i.e., pre‐chemotherapy vs. post‐chemotherapy); both tests were two‐tailed. An alpha of 0.05 was used, whereby a *p* < 0.05 was considered significant and denoted with an asterisk in graphs of comparisons. Boxplots were used to present the median, spread, and potential outliers according to the 1.5 × interquartile range method, additionally, the mean is overlaid as a white box with white dashed error bars representing ±1 standard error. Multiple comparisons were adjusted via the false discovery rate (FDR) adjusting Benjamini‐Hochberg (BH) procedure [19]. An FDR rate of 5% (*q* = 0.05) was used, and the number of hypotheses tested for each comparison was 13 (m = 13). BH adjusted *p* values less than the FDR rate of 5% within a comparison group indicate these differences are significant given that it is expected that 5% of the positive findings within that comparison group to be false positives. Adjusted *p* values can be seen in Table S2. Boxplots were generated with R (R version 4.3.2 (2023‐10‐31), https://www.r‐project.org/) using the ggplot2 package and arranged in multi‐panel figures using the ggarrange package. Two‐sample and paired *t*‐tests were run using Excel (version 2508) through the Analysis ToolPak add‐in. Excel was also used to manually calculate FDR‐adjusted *p* values. Unless specified, the cancer group as a whole included both anemic and non‐anemic patients in analysis, due to group size constraints.

## 3 Results

The following figures highlight only significant findings related to aggregability, deformability, and elasticity between comparison groups.

### 3.1 Clinical and Demographic Features of Cancer Cohort

Demographic data of the 110 patients; 62 with breast cancer, 25 ovarian and 24 endometrial cancer, along with controls, are presented in Tables S3.1, S3.2, and S3.3. The average age of patients was 62.4 years. The majority of patients had a BMI of ≥ 30, were post‐menopausal, nonsmokers, and received adjuvant chemotherapy. Prior to chemotherapy, 77.08% had no anemia, whereas after chemotherapy, anemia increased in both frequency and severity with only 60.42% remaining non‐anemic.

Despite the difference in mean age between the controls and cancer patients, analysis of MIZAR parameters between age quartiles of controls showed no significant difference in any parameter between quartiles. Regarding CBC parameters, hemoglobin levels in females remain relatively steady throughout adulthood [20].

### 3.2 Cancer Patients Exhibit Altered CBC Results and RBC Biomechanics

Between controls and patients with cancer pre‐chemotherapy, two CBC parameters were significantly different. Mean corpuscular hemoglobin concentration (MCHC) was significantly lower and mean platelet volume (MPV) was significantly higher in cancer patients than in healthy controls.

**FIGURE 1:**
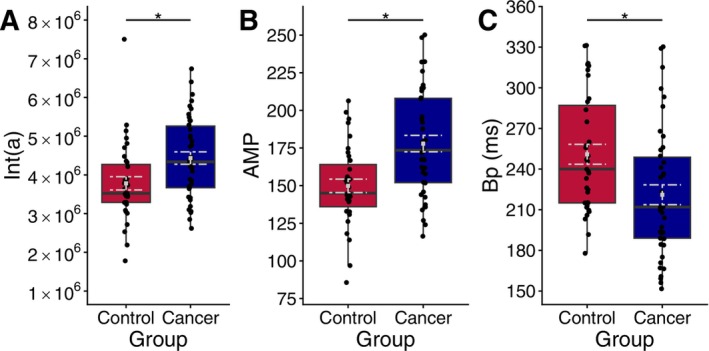
Aggregation integral (Int(a), A), amplitude (AMP, B), and base point (Bp, C) for healthy controls compared to cancer patients. Int(a) gives the magnitude of the aggregation phenomena, AMP is proportional to aggregation, and Bp represents the time for cells to regain their shape (lower Bp indicates more elastic cells). *Represents *p* < 0.05 between groups. Gray square indicates mean, dotted lines indicate standard error (SE). Dark solid line within each box represents median, the whiskers extend to the maximum and minimum values that aren't outliers, based on the 1.5×IQR rule.

Comparing cancer patients (pre‐chemotherapy treatment) with healthy controls demonstrated that RBC aggregation was significantly higher in the patients, and their cells were more elastic (Figure 1). That is, their shape recovery after deformation was faster than in the controls. Table S2 shows comparative analysis of controls, cancer patients pre‐ and post‐chemotherapy, anemic and non‐anemic, and highlights significant alterations in the various RBCs' biomechanical parameters in all patient groups.

### 3.3 Anemia in Cancer Patients Is Associated With Changes in RBC Biomechanics

**FIGURE 2:**
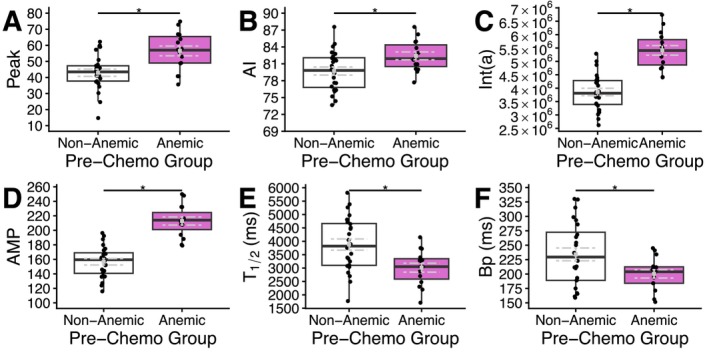
Redistribution peak (Peak, A), aggregation index (AI, B), aggregation integral (Int(a), C), amplitude (AMP, D), half time (T_1/2_, E), and base point (Bp, F) for non‐anemic and anemic cancer patients. Peak is proportional to deformability, AI and Int(a) give the speed and magnitude of the aggregation phenomena, AMP represents aggregation, T_1/2_ indicates the speed of aggregation (lower time represents faster aggregation), and Bp represents the time for cells to regain their shape (lower Bp indicates more elastic cells). *Represents *p* < 0.05 between groups. Gray square indicates mean, dotted lines indicate standard error (SE). Dark solid line within each box represents median, the whiskers extend to the maximum and minimum values that are not outliers, based on the 1.5×IQR rule.

Anemic cancer patients had several differences in their RBC biomechanics when compared with non‐anemic patients. Namely, anemic patients' RBCs were more deformable than non‐anemic patients (Figure 2). Additionally, anemic patients exhibited increased RBC aggregation, faster aggregation, and more elastic RBCs than non‐anemic patients (Figure 2).

### 3.4 Post‐Chemotherapy, Patients Exhibit Similar Biomechanical Changes

**FIGURE 3:**
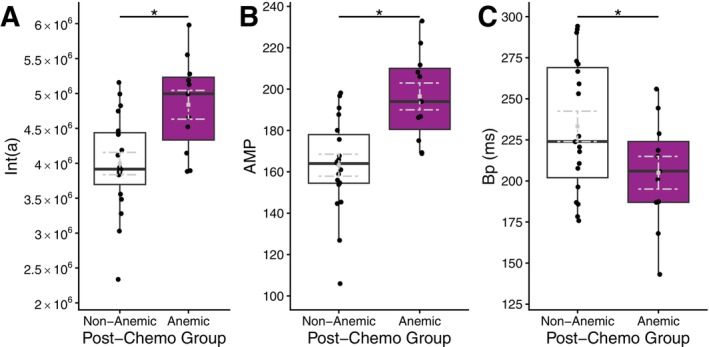
Aggregation integral (Int(a), A), amplitude (AMP, B), and base point (Bp, C) for non‐anemic and anemic cancer patients after undergoing chemotherapy. Int(a) gives the magnitude of the aggregation phenomena, AMP represents aggregation, and Bp represents the time for cells to regain their shape (lower Bp indicates more elastic cells). *Represents *p* < 0.05 between groups. Gray square indicates mean, dotted lines indicate standard error (SE). Dark solid line within each box represents median, the whiskers extend to the maximum and minimum values that aren't outliers, based on the 1.5×IQR rule.

When comparing post‐chemotherapy anemic versus non‐anemic patients, similar results were seen. Post‐chemotherapy, anemic patients' RBC changes exhibited evidence of higher aggregation and elasticity than their non‐anemic counterparts (Figure 3).

### 3.5 Chemotherapy Is Associated With CBC Changes and Increased RBC Elasticity and Decreased Deformability

After chemotherapy, cancer patients exhibited lower RBC count, hemoglobin, hematocrit, platelets, and MPV, with higher red cell distribution width (RDW, a marker of inflammation) than before chemotherapy.

**FIGURE 4:**
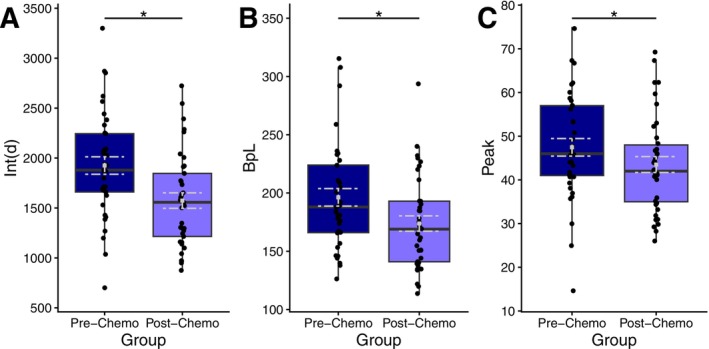
Distribution integral (Int(d), A), base point low (BpL, B), and redistribution peak (Peak, C) for cancer patients before and after undergoing chemotherapy. Int (d) is inversely proportional to elasticity, BpL represents the time for cells to regain their shape (lower BpL indicates more elastic cells), and peak is proportional to deformability.

Additionally, after chemotherapy, cancer patients displayed higher RBC elasticity and lower deformability than before chemotherapy (Figure 4).

### 3.6 Chemotherapy Is Associated With RBC Biomechanical Changes in Anemic Patients

**FIGURE 5:**
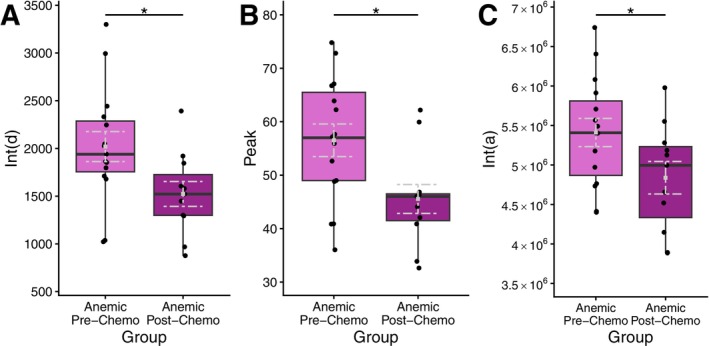
Distribution integral (Int(d), A), redistribution peak (Peak, B), and aggregation integral (Int(a), C) for anemic cancer patients before and after undergoing chemotherapy. Int(d) is inversely proportional to elasticity, peak is proportional to deformability, and Int(a) gives the magnitude of the aggregation phenomena. *Represents *p* < 0.05 between groups. Gray square indicates mean, dotted lines indicate standard error (SE). Dark solid line within each box represents median, the whiskers extend to the maximum and minimum values that are not outliers, based on the 1.5×IQR rule.

Post‐chemotherapy, anemic cancer patients exhibited greater RBC elasticity while having lower deformability and aggregation when compared with anemic cancer patients before chemotherapy treatment (Figure 5).

## 4 Discussion

### 4.1 Cancer Patients Exhibit Altered RBC Biomechanics

This study shows based on automated syllectometry, women's cancer patients have increased RBC aggregation compared to healthy controls. There has been little research documenting impaired RBC biomechanics in cancer. However, one study investigating patients with various cancer types (both males and females) before any treatment found that they displayed increased RBC aggregation [21]. RBC aggregation is indicative of inflammation, which is also well‐documented as a hallmark of cancer [22]. There is currently no known research on RBC elasticity specifically in cancer (as opposed to deformability). The fact that patients displayed higher RBC elasticity calls for further research to confirm these findings. A potential mechanism by which cancer increases RBC elasticity is through cytoskeletal remodeling induced by ATP depletion. Inflammatory states such as sepsis are known to deplete ATP in RBCs, and cancer's chronic inflammation may have similar effects [23]. ATP‐depleted RBCs have been shown to recover faster (greater elasticity) than intact cells as the elastic component becomes more dominant relative to the viscous component [24]. Biophysical studies suggest this occurs because ATP hydrolysis is required for spectrin–actin junction dissociation; with depleted ATP, the cytoskeleton becomes rigid and cannot slowly remodel, causing RBCs to recover their shape more quickly after deformation [25]. While this mechanistic connection is plausible, it remains theoretical and must be interpreted with caution.

### 4.2 Anemia in Cancer Patients Is Associated With Changes in RBC Biomechanics

Previous research has found decreased RBC deformability in patients with solid tumors, but especially those with anemia [26]. Several studies have also found decreased RBC deformability in patients with iron‐deficiency anemia compared to healthy controls [27, 28, 29]. Reduced RBC deformability has been observed in patients with megaloblastic anemia [30]. One study investigating proteinuric chronic kidney disease found that reduced RBC deformability may contribute to the development of anemia in patients with the disease [31]. Given these findings, the consensus appears to be that anemia is associated with reduced RBC deformability.

On the contrary, in the present study, anemic cancer patients' RBCs were significantly more deformable than in non‐anemic patients. A proposed theory is that the lower hemoglobin concentration in anemic patients may lead to decreased RBC cytoplasmic viscosity, which leads to higher deformability [32]. Another theory is that since anemia causes the bone marrow to release more immature RBCs (reticulocytes), deformability may be higher simply due to the higher ratio of younger RBCs; RBC deformability decreases as RBCs age [33]. The higher elasticity found is likely related to these findings, as lower cytoplasmic viscosity and higher deformability would reduce viscous resistance and allow the elastic component to dominate, leading to faster recovery [24]. However, this requires further investigation; the theory of younger RBCs may explain the results related to deformability and elasticity but does not support the results related to aggregation.

This study also demonstrated that anemic cancer patients exhibited increased aggregation compared to non‐anemic patients. This suggests that inflammation may also be increased and confirms findings from past studies. One study found that anemic patients with head and neck cancers had elevated CRP (a marker of inflammation) compared to non‐anemic patients [34]. Another study found that anemic solid cancer patients had higher concentrations of inflammatory markers CRP and neopterin than non‐anemic patients [35]. Another study found that in patients with various cancers, hemoglobin concentration was inversely correlated with inflammatory markers, specifically CRP, fibrinogen, IL‐6, TNFα, IL‐1β, ferritin, hepcidin, and ROS [36].

The present study found that anemic cancer patients displayed increased and faster RBC aggregation compared to non‐anemic patients. There is currently no known research that directly compares RBC aggregation in anemic cancer patients to non‐anemic cancer patients. However, one study found that when anemic cancer patients were treated with Epoetin Alfa (EA), their RBC aggregability decreased. Once the anemia was corrected, however, further EA treatment increased aggregation, resembling the response seen in healthy controls. It has been proven experimentally that Ca^2+^ plays an essential role in cell adhesion and aggregation; Ca^2+^ entry into RBCs activates calmodulin, which stimulates membrane adhesion receptors and promotes fibrinogen binding to integrin‐like receptors on the RBC membrane, thereby intensifying aggregation [37]. Further investigation into this mechanism and how it may relate to the results of this study is needed.

### 4.3 Chemotherapy Is Associated With Increased RBC Elasticity and Decreased Deformability

There are several theories that may explain why cancer patients post‐chemotherapy may have higher RBC elasticity than pre‐chemotherapy. Firstly, one study found an increase in membrane fluidity in the RBCs of patients with breast cancer receiving chemotherapy [38]. Increased membrane fluidity may lead to faster shape recovery after deformation (elasticity). Another theory is that lower saturated fatty acids in the RBC membranes allow for higher membrane elasticity. Several studies have found that various types of cancer patients receiving chemotherapy have lower saturated fatty acids in their RBC membranes [38].

The finding of this study that cancer patients had decreased RBC deformability after undergoing chemotherapy confirms data from past studies. One study found that treatment with paclitaxel resulted in reduced RBC deformability [39]. Another study found that chemotherapy drugs paclitaxel and docetaxel induced a stomatocytic shape transformation in RBCs, which resulted in decreased deformability [40].

### 4.4 Chemotherapy Is Associated With RBC Biomechanical Changes in Anemic Patients

There is currently no known research comparing RBC biomechanical changes in anemic cancer patients before and after chemotherapy. However, the reasons listed above regarding chemotherapy increasing RBC elasticity and decreasing deformability can likely be applied to the comparison between anemic cancer groups. Further research is needed to confirm this, and to understand why anemic patients displayed decreased aggregability after undergoing chemotherapy.

### 4.5 Impact of Anemia

The following RBC biomechanical parameters: aggregation and elasticity were significantly different between pre‐chemotherapy patients and controls, pre‐chemotherapy anemic patients and controls, and anemic and non‐anemic pre‐chemotherapy patients. Interestingly, the anemic and non‐anemic patients significantly differ from each other in these same parameters, thus we can speculate that the significant differences seen between pre‐chemotherapy patients and controls may be due to the anemia itself rather than the cancer.

In post‐chemotherapy patients, the following parameters: aggregation and elasticity were significantly different in anemic patients compared to controls and were significantly different between anemic and non‐anemic patients. These were the same parameters as in the pre‐chemotherapy comparison groups mentioned above. Once again, this data suggests that the differences in these parameters may be a result of the anemia rather than the cancer. The fact that there are two additional parameters that significantly differ post‐chemotherapy suggests that chemotherapy may exaggerate the effects of anemia on these parameters (if that is indeed the cause of the differences seen), possibly because of the increased frequency and severity of anemia experienced in patients after undergoing chemotherapy.

### 4.6 BMI Considerations

The high BMI among patients was a notable characteristic of the cohort and may represent a potential confounding factor when interpreting RBC rheological findings. Obesity is associated with chronic low‐grade inflammation and altered hemorheological properties, including increased RBC aggregation and reduced deformability [41], Therefore, elevated BMI in the cohort may have contributed, at least in part, to the observed rheological alterations, independent of the effects of cancer or anemia. These findings should be interpreted with this potential influence in mind.

### 4.7 Future Research

Further studies should examine RBCs biomechanics in non‐cancer patients with anemia to further elucidate if these changes are specific to cancer‐associated anemia. Additionally, further large studies should compare RBC biomechanics between different cancer types and examine the effects of cancer stage and types of chemotherapy and other patients' factors such as age, BMI, and comorbidities.

## 5 Conclusions

This study demonstrates novel observations that cancer is associated with changes in RBC biomechanical properties, specifically increased aggregation and elasticity. Chemotherapy is also associated with biomechanical changes: RBC elasticity was even higher after chemotherapy, and deformability was decreased.

This data suggests that anemia in cancer patients may be linked to those changes, since anemic patients, whether pre‐ or post‐chemotherapy, displayed increased aggregation and elasticity. This highlights a new angle in cancer‐associated anemia beyond quantitative RBC analyses.

We recognize that these early observations add to the complexity of our understanding of cancer‐associated anemia, a global health issue, and highlight an important notion that conventional tests may not be sufficient to evaluate anemia in cancer and under cancer therapies; both the quantity and quality of RBCs must be considered. Further research is needed to examine the mechanisms by which cancer and cancer therapies may impair RBC biomechanics, RBC function, and how these may impact patient care.

## 6 Perspectives

Women with gynecologic and breast cancers exhibit altered RBC biomechanical properties that are further potentiated by anemia and chemotherapy. Findings from this study suggest that conventional hematologic measurements alone may not fully capture the functional impairments in RBCs that could contribute to inflammation, microcirculatory dysfunction, and patient morbidity in cancer‐associated anemia. Better understanding of RBC biomechanics may support the future development of novel biomarkers and more targeted treatment approaches for cancer patients.

## Funding

Funding for this work was provided by the Queen's University Department of Biomedical and Molecular Sciences Teaching Assistantship program, Queen's University Graduate Award program, and ALCOR Scientific LLC.

## Ethics Statement

This study was approved by the Queen's University Health Sciences and Affiliated Teaching Hospitals Research Ethics Board (HSREB) and the St. Lawrence College Research Ethics Board.

## Consent

Written informed consent was obtained from all participants prior to inclusion in the study.

## Conflicts of Interest

The authors declare no conflicts of interest.

## Supporting information


**Figure S1:** Characteristic shape of a syllectogram obtained via the MIZAR analyzer, with the parameters related to biomechanical properties outlined according to the property they relate to; aggregation index (AI), end transmission (ED), aggregation integral (int(a)), amplitude (AMP), optical transmission during flow (OTF), distribution index (DI), redistribution peak (peak), optical transmission (OT), and distribution integral (int(d)) [5].
**Table S1:** Explanation of parameters collected from the MIZAR analyzer [5] (ALCOR Scientific LLC.). Orange shading indicates relation to elasticity, green shading indicates relation to deformability, and blue shading indicates relation to aggregability.
**Table S2:** *p* Values and adjusted *p* values for each comparison, adjusted using the Benjamini‐Hochberg procedure. Bolded *p* values indicate a *p* value less than 0.05, where an alpha of 0.05 was used for unadjusted *p* values, and a false discovery rate of 0.05 was used for adjusted *p* values.
**Table S3.1:** Cancer patient (*n* = 110) demographics and clinical data. N/A indicates either no associated finding or no data available.
**Table S3.2:** Cancer patient (*n* = 110) demographics/clinical data, pt. 2. N/A indicates either no associated finding or no data available.
**Table S3.3:** Control group (*n* = 35) demographics.

## Data Availability

Dataset is available upon request.
