# Supplementary material for: Evaluation of Red Blood Cell Biomechanics in the Setting of Cancer‐Associated Anemia and Chemotherapy
Source: Microcirculation. 2026 Jun 26;33(5):e70074. doi: 10.1111/micc.70074 (PMC13309682; doi:10.1111/micc.70074)
Supplement: Supplementary file 1 — Figure S1: Characteristic shape of a syllectogram obtained via the MIZAR analyzer, with the parameters related to biomechanical properties outlined according to the property they relate to; aggregation index (AI), end transmission (ED), aggregation integral (int(a)), amplitude (AMP), optical transmission during flow (OTF), distribution index (DI), redistribution peak (peak), optical transmission (OT), and distribution integral (int(d)) [5]. Table S1: Explanation of parameters collected from the MIZAR analyzer [5] (ALCOR Scientific LLC.). Orange shading indicates relation to elasticity, green shading indicates relation to deformability, and blue shading indicates relation to aggregability. Table S2: p Values and adjusted p values for each comparison, adjusted using the Benjamini‐Hochberg procedure. Bolded p values indicate a p value less than 0.05, where an alpha of 0.05 was used for unadjusted p values, and a false discovery rate of 0.05 was used for adjusted p values. Table S3.1: Cancer patient (n = 110) demographics and clinical data. N/A indicates either no associated finding or no data available. Table S3.2: Cancer patient (n = 110) demographics/clinical data, pt. 2. N/A indicates either no associated finding or no data available. Table S3.3: Control group (n = 35) demographics. [file MICC-33-e70074-s001.docx]

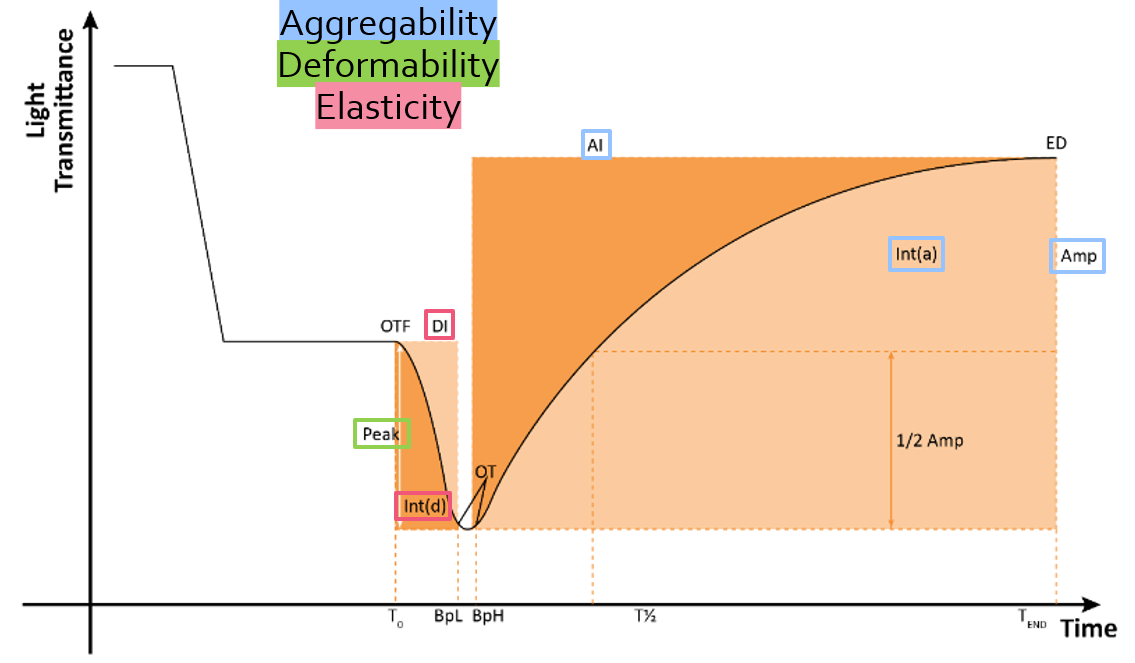


**Figure S1: Characteristic shape of a syllectogram obtained via the MIZAR^TM^ analyzer, with the parameters related to biomechanical properties outlined according to the property they relate to; aggregation index (AI), end transmission (ED), aggregation integral (int(a)), amplitude (AMP), optical transmission during flow (OTF), distribution index (DI), redistribution peak (peak), optical transmission (OT), and distribution integral (int(d))** ^5^**.**

Table S1: Explanation of parameters collected from the MIZAR^®^ analyzer^5^ALCOR® Scientific LLC.). Orange shading indicates relation to elasticity, green shading indicates relation to deformability, and blue shading indicates relation to aggregability.

| **MIZAR**^®^ **Parameter** | **Abbreviation** | **Explanation** |
| --- | --- | --- |
| Distribution Index | DI | Ratio between Int(d) and the area of the abstract rectangle. DI is a calculated parameter which is inversely proportional to elasticity. |
| Distribution Integral | Int(d) | The area under the RBCs Distribution Stage of the Syllectogram. This parameter is inversely proportional to elasticity. |
| Redistribution Peak | Peak | This parameter is directly proportional to RBC deformability and calculated as the difference between OTF and OT. |
| Aggregation Index | AI | Ratio between Int(a) and the area of the abstract rectangle. AI is a calculated parameter that is proportional to inflammation (aggregation) |
| Aggregation Integral | Int(a) | Area under the RBCs Aggregation Stage of the Syllectogram, a parameter proportional to inflammation (aggregation) |
| Amplitude | AMP | The amplitude of the kinetic, obtained by the difference from ED and OT (ED-OT), resulting from RBCs aggregation. |
| Base Point | BP | Central time between BpL and BpH, can be considered as average time for shape recovery |
| Base Point Low | BpL | First point in time on which minimum optical value has been detected. Time to complete RBCs shape recovery |
| Base Point High | BpH | Last point in time before RBCs start the aggregation process |
| Half Time | T_1/2_ | Provides information on the speed of the aggregation kinetics and is the time necessary to reach half of the amplitude (AMP). Gives information of the speed of aggregation. |
| Optical Transmission | OT | The minimum optical transmission value after the pumping system is stopped and shear stress application is removed. The sample is now in stasis. After the shear stress is removed, RBCs regain their natural shape and redistribute uniformly in the available volume. This is inversely correlated to the Hematocrit (Hct) |
| Optical Transmission During Flow | OTF | The value attained during shear stress application, when the sample is transported into the reading cell |
| End Transmission | ED | Plateau |

**Table S2: P-values and adjusted p-values for each comparison, adjusted using the Benjamini-Hochberg procedure.** Bolded p-values indicate a p-value less than 0.05, where an alpha of 0.05 was used for unadjusted p-values, and a false discovery rate of 0.05 was used for adjusted p-values.

|  | Ctrl vs. Pre-Chemo | | | | | |
| --- | --- | --- | --- | --- | --- | --- |
|  | n_ctrl_=35, n_pre_=42 | | | | | |
| MIZAR™ Parameters | Ctrl Mean (SD) | Pre Mean (SD) | p-value | | Adj p-value | |
| AI | 79.4 (4) | 80.5 (3.6) | 0.202 | | 0.292 | |
| AMP | 149.9 (26.5) | 177.9 (35.3) | **<0.001** | | **0.002** | |
| Bp (ms) | 250.9 (43.5) | 221 (47.5) | **0.005** | | **0.017** | |
| BpH (ms) | 294.2 (64.6) | 249.6 (62.6) | **0.003** | | **0.017** | |
| BpL (ms) | 208 (52.1) | 193 (45.1) | 0.186 | | 0.292 | |
| DI | 20.7 (3.9) | 21.7 (6.1) | 0.388 | | 0.425 | |
| ED | 1417.1 (44.5) | 1457.4 (72.6) | **0.004** | | **0.017** | |
| Int(a) | 3.8×10⁷ (1.0×10⁷) | 4.4×10⁷ (1.0×10⁷) | **0.007** | | **0.018** | |
| Int(d) | 2013.6 (516.8) | 1908.4 (554.8) | 0.392 | | 0.425 | |
| OT | 1267.2 (26.4) | 1279.5 (40.8) | 0.115 | | 0.249 | |
| OTF | 1315.8 (34) | 1327.5 (50.1) | 0.229 | | 0.297 | |
| Peak | 48.6 (10.4) | 48 (12.6) | 0.814 | | 0.814 | |
| T_1/2_ (ms) | 3970.4 (1176) | 3620.4 (1028.4) | 0.173 | | 0.292 | |
|  | Pre-Chemo vs. Post-Chemo | | | | |  |
|  | n_pre/post_=37 | | | | |  |
| MIZAR™ Parameters | Pre Mean (SD) | Post Mean (SD) | p-value | Adj p-value | |  |
| AI | 80.4 (3.5) | 80.1 (3.7) | 0.516 | 0.673 | |  |
| AMP | 176.3 (33.2) | 180.8 (28.5) | 0.149 | 0.336 | |  |
| Bp (ms) | 223.1 (49.1) | 214.9 (39.7) | 0.181 | 0.336 | |  |
| BpH (ms) | 250.4 (64.5) | 256.3 (58.1) | 0.518 | 0.673 | |  |
| BpL (ms) | 196.3 (45.8) | 173.8 (39.9) | **0.009** | 0.062 | |  |
| DI | 21.8 (6.4) | 21.3 (3.5) | 0.658 | 0.738 | |  |
| ED | 1453.5 (71.1) | 1464 (62.9) | 0.108 | 0.336 | |  |
| Int(a) | 4.4×10⁷ (9.6×10⁶) | 4.5×10⁷ (8.3×10⁶) | 0.340 | 0.552 | |  |
| Int(d) | 1925.2 (531.4) | 1573.9 (471.6) | **<0.001** | **0.008** | |  |
| OT | 1277.2 (41) | 1283.2 (38.2) | 0.156 | 0.336 | |  |
| OTF | 1324.7 (49.9) | 1326.8 (44.6) | 0.681 | 0.738 | |  |
| Peak | 47.5 (12.2) | 43.5 (11.1) | **0.030** | 0.131 | |  |
| T_1/2_ (ms) | 3651.9 (998.4) | 3634.5 (1097.5) | 0.896 | 0.896 | |  |

|  | Pre-Chemo: Non-Anemic vs. Anemic | | | | | | |  |
| --- | --- | --- | --- | --- | --- | --- | --- | --- |
|  | n_preNA_=24, n_preA_=15 | | | | | | |  |
| MIZAR™ Parameters | PreNA Mean (SD) | PreA Mean (SD) | | p-value | | Adj p-value | |  |
| AI | 79.7 (3.5) | 82.4 (2.7) | | **0.011** | | **0.016** | |  |
| AMP | 156.7 (21.6) | 212.9 (21.7) | | **<0.001** | | **<0.001** | |  |
| Bp (ms) | 234.1 (54.7) | 200.3 (28) | | **0.016** | | **0.020** | |  |
| BpH (ms) | 267.1 (71.3) | 220 (34) | | **0.009** | | **0.014** | |  |
| BpL (ms) | 201.6 (52.4) | 181.1 (33.8) | | 0.145 | | 0.157 | |  |
| DI | 23.1 (7.4) | 19.8 (2.7) | | 0.055 | | 0.065 | |  |
| ED | 1408.5 (31.8) | 1535.3 (46.2) | | **<0.001** | | **<0.001** | |  |
| Int(a) | 3.9×10⁷ (6.8×10⁶) | 5.4×10⁷ (6.9×10⁶) | | **<0.001** | | **<0.001** | |  |
| Int(d) | 1878 (539) | 2019.3 (608.7) | | 0.468 | | 0.468 | |  |
| OT | 1251.8 (17.5) | 1322.4 (28.9) | | **<0.001** | | **<0.001** | |  |
| OTF | 1294.8 (25.1) | 1378.9 (36.6) | | **<0.001** | | **<0.001** | |  |
| Peak | 42.9 (10.9) | 56.5 (11.8) | | **0.001** | | **0.003** | |  |
| T_1/2_ (ms) | 3878.5 (1022.6) | 3015.5 (654.1) | | **0.003** | | **0.005** | |  |
|  | Post-Chemo: Non-Anemic vs. Anemic | | | | | | | |
|  | n_postNA_=19, n_postA_=11 | | | | | | | |
| MIZAR™ Parameters | PostNA Mean (SD) | | PostA Mean (SD) | | p-value | | Adj p-value | |
| AI | 79.4 (3.5) | | 79.8 (3.6) | | 0.750 | | 0.750 | |
| AMP | 163.2 (23.2) | | 196.5 (21.5) | | **<0.001** | | **0.003** | |
| Bp (ms) | 233.2 (40.3) | | 205 (33) | | **0.048** | | 0.092 | |
| BpH (ms) | 282.6 (60.6) | | 243.2 (43.6) | | **0.049** | | 0.092 | |
| BpL (ms) | 184.2 (43.6) | | 167.3 (40.2) | | 0.293 | | 0.423 | |
| DI | 21.8 (4.1) | | 20.3 (3) | | 0.259 | | 0.421 | |
| ED | 1425.5 (39.6) | | 1506.5 (49.9) | | **<0.001** | | **0.003** | |
| Int(a) | 4.0×10⁷ (7.0×10⁶) | | 4.8×10⁷ (6.8×10⁶) | | **0.004** | | **0.010** | |
| Int(d) | 1669.3 (522.3) | | 1523.5 (431.3) | | 0.418 | | 0.502 | |
| OT | 1262.3 (19.2) | | 1310.1 (31.8) | | **<0.001** | | **0.003** | |
| OTF | 1304.8 (26.4) | | 1355.6 (35.5) | | **<0.001** | | **0.003** | |
| Peak | 42.6 (10.7) | | 45.5 (9) | | 0.424 | | 0.502 | |
| T_1/2_ (ms) | 3893.8 (1099.2) | | 3654 (968.4) | | 0.540 | | 0.585 | |

|  | Anemic: Pre-Chemo vs. Post-Chemo | | | |
| --- | --- | --- | --- | --- |
|  | n_preA_=15, n_postA_=11 | | | |
| MIZAR™ Parameters | PreA Mean (SD) | PostA Mean (SD) | p-value | Adj p-value |
| AI | 82.4 (2.7) | 79.8 (3.6) | 0.062 | 0.166 |
| AMP | 212.9 (21.7) | 196.5 (21.5) | 0.069 | 0.166 |
| Bp (ms) | 200.3 (28) | 205 (33) | 0.708 | 0.708 |
| BpH (ms) | 220 (34) | 243.2 (43.6) | 0.160 | 0.231 |
| BpL (ms) | 181.1 (33.8) | 167.3 (40.2) | 0.367 | 0.434 |
| DI | 19.8 (2.7) | 20.3 (3) | 0.659 | 0.708 |
| ED | 1535.3 (46.2) | 1506.5 (49.9) | 0.150 | 0.231 |
| Int(a) | 5.4×10⁷ (6.9×10⁶) | 4.8×10⁷ (6.8×10⁶) | **0.047** | 0.166 |
| Int(d) | 2019.3 (608.7) | 1523.5 (431.3) | **0.023** | 0.149 |
| OT | 1322.4 (28.9) | 1310.1 (31.8) | 0.323 | 0.419 |
| OTF | 1378.9 (36.6) | 1355.6 (35.5) | 0.117 | 0.217 |
| Peak | 56.5 (11.8) | 45.5 (9) | **0.013** | 0.149 |
| T_1/2_ (ms) | 3015.5 (654.1) | 3654 (968.4) | 0.077 | 0.166 |

Table S3.1. Cancer patient (n = 110) demographics and clinical data. N/A indicates either no associated finding or no data available.

| **Age (mean ± SD)** | | 62.38 ± 12.31 |
| --- | --- | --- |
| **BMI** | | |
| Healthy (18.5-24.9) | | 39 (35.45%) |
| Overweight (25-29.9) | | 25 (22.73%) |
| Obese (≥ 30) | | 44 (40.00%) |
| N/A | | 2 (1.82%) |
| **Menopausal Status** | | |
| Pre | | 20 (18.18%) |
| Post | | 90 (81.82%) |
| **Smoking Status** | | |
| Current | | 14 (12.73%) |
| Remote | | 29 (26.36%) |
| Never | | 65 (59.09%) |
| N/A | | 2 (1.82%) |
| **Comorbidities** | | |
| Diabetes | | 16 (14.55%) |
| Hypertension | | 37 (33.64%) |
| Coronary Artery Disease | | 4 (3.64%) |
| Dyslipidemia | | 20 (18.18%) |
| Aortic Stenosis | | 1 (0.91%) |
| N/A | | 59 (53.64%) |
| **Hematological Findings** | | |
| Pancytopenia | | 7 (7.07%) |
| Thrombocytopenia | | 9 (9.09%%) |
| Neutropenia | | 3 (3.03%) |
| Anemia | | 3 (2.73%) |
| N/A | | 91 (82.73%) |
| **Cancer Type** | | |
| Breast | | 62 (56.36%) |
| Ovarian | | 25 (22.73%) |
| Endometrial | | 24 (21.82%) |
| **Breast Cancer Histology** | | |
| Ductal | 47 (75.81%) | |
| Lobular | 9 (14.52%) | |
| Metaplastic | 3 (4.84%) | |
| Papillary | 4 (6.45%) | |
| Mucinous | 1 (1.61%) | |
| N/A | 2 (3.23%) | |

Table S3.2. Cancer patient (n = 110) demographics/clinical data, pt 2. N/A indicates either no associated finding or no data available.

| **Ovarian Cancer Histology** | | |
| --- | --- | --- |
| Serous | 19 (76.00%) |  |
| Mucinous | 0 (0.00%) |  |
| Clear Cell | 2 (8.00%) |  |
| Endometroid | 1 (4.00%) |  |
| Adenocarcinoma | 2 (8.00%) |  |
| N/A | 4 (16.00%) |  |
| **Endometrial Cancer Histology** | | |
| Endometroid Adenocarcinoma | 15 (62.50%) |  |
| Papillary Serous | 5 (20.83%) |  |
| Clear Cell | 2 (8.33%) |  |
| Carcinosarcoma | 0 (0.00%) |  |
| Sarcoma | 1 (4.17%) |  |
| N/A | 1 (4.17%) |  |
| **Cancer Stage** | | |
| Palliative (Stage IV) | 25 (22.73%) |  |
| Advanced (Stage III) | 33 (30.00%) |  |
| Early (Stage I-II) | 49 (44.55%) |  |
| N/A | 3 (2.73%) |  |
| **Chemotherapy** | | |
| Adjuvant | 63 (57.27%) |  |
| Neoadjuvant | 41 (37.27%) |  |
| Both | 6 (5.45%) |  |
| **Type of Chemotherapy** | | |
| Non-Platinum Based | 53 (48.18%) |  |
| Platinum Based | 57 (51.82%) |  |
| **Anemia (n=96)** | | |
| **Pre-Chemotherapy** | | |
| None | 74 (77.08%) |  |
| Grade I | 20 (20.83%) |  |
| Grade II | 1 (1.04%) |  |
| Grade III | 1 (1.04%) |  |
| Grade IV | 0 (0.00%) |  |
| **Post-Chemotherapy** | | |
| None | 58 (60.42%) |  |
| Grade I | 30 (31.25%) |  |
| Grade II | 6 (6.25%) |  |
| Grade III | 2 (2.08%) |  |
| Grade IV | 0 (0.00%) |  |

Table S3.3. Control group (n = 35) demographics

| **Age ± SD** | 27.71 ± 9.55 |
| --- | --- |
| **Smoking Status** | |
| Cannabis | 4 (11.43%) |
| Tobacco | 0 (0.00%) |
| None | 31 (88.57%) |
